# α-MSH and melanocortin receptors at early ontogeny in European sea bass (*Dicentrarchus labrax*, L.)

**DOI:** 10.1038/srep46075

**Published:** 2017-04-05

**Authors:** A. Tsalafouta, M. Gorissen, T. N. M. Pelgrim, N. Papandroulakis, G. Flik, M. Pavlidis

**Affiliations:** 1 Hellenic Center for Marine Research, Institute of Marine Biology, Biotechnology and Aquaculture, P.O. Box 2214, Heraklion, Crete, Greece; 2 University of Crete, Department of Biology, P.O. Box 2208, GR-714 09, Heraklion, Crete, Greece; 3 Radboud University Nijmegen, Institute for Water and Wetland Research, Department of Animal Ecology and Physiology, Heyendaalseweg 135, 6525AJ, The Netherlands

## Abstract

Temporal patterns of whole-body α-MSH concentrations and of transcripts of melanocortin receptors during early development as well as the endocrine response (α-MSH, cortisol, MCR mRNAs) to stress at the end of the larval period were characterized in *Dicentrarchus labrax*. Immunohistochemistry showed α-MSH positive cells in the pituitary *pars intermedia* in all stages examined. As development proceeds, α-MSH content gradually increases; mRNA levels of *mc2r* and *mc4r* remain low until first feeding where peak values are observed. *Mc1r* expression was constant during development, *pomc* mRNA levels remain low until the stage of flexion after which a significant increase is observed. At the stage of the formation of all fins, whole-body cortisol and α-MSH concentrations responded with peak values at 2 h post stress. Additionally, the stress challenge resulted in elevated transcript levels of *pomc, mc2r* and *mc4r* but not in *mc1r*, with a pattern characterized by peak values at 1 h post stress and a strong correlation with whole body α-MSH concentrations was found. Our data provide for the first time a view on the importance of the α-MSH stress response in early development of European sea bass, an additional and relatively poorly understood signal involved in the stress response in teleosts.

In fish, stress leads to the activation of the hypothalamic-pituitary-interrenal (HPI) axis, and stimulates the pituitary corticotropes in the *pars distalis* and the melanotropes in the *pars intermedia* to synthesize and secrete pro-opiomelanocortin (POMC)-derived peptides involved in the mediation and regulation of the stress response^1^,^2^. The pituitary gland is the major site of *pomc* expression. The gene is translated into a precursor protein, from which, among others, in the pars distalis adrenocorticotropic hormone (ACTH) and in the pars intermedia alpha-melanocyte stimulating hormone (α-MSH) are derived^3^. In European sea bass (*Dicentrarchus labrax*, L.) a single form of a functional *pomc* gene has been cloned and characterized^4^. Melanocortins exert their physiological role by binding to a family of specific G protein-coupled receptors (GPCRs) that positively couple to adenylyl cyclase. Tetrapod species have five melanocortin receptors (MC1R-MC5R), although in teleost fish the number of receptors differs^5^,^6^. The MC2R is specifically activated by ACTH, while the other MCRs can be activated by α-MSH as well as ACTH^7^.

During HPI axis activation, ACTH secreted by the corticotropes is a key regulator of the acute stress response as it stimulates the interrenal cells via MC2R and results in the synthesis and secretion of cortisol which targets a plethora of tissues, if not all cells in an organism^1^,^7^,^8^. Relatively few studies have focused on the function and characterization of MC2R in fish^9^,^10^,^11^,^12^,^13^ and a recent study conducted in European sea bass demonstrated a negative feedback by cortisol on MC2R expression in a chronic stress paradigm^14^.

There are 4 MCRs which convey MSH signals: MC1R, MC3R, MC4R and MC5R. Interaction of α-MSH and the MC1R plays a key role in the control of the pigmentation. For instance, mutations in the MC1R are responsible for reduced melanization. MC3R and MC4R are considered ‘brain receptors’; the expression of the MC4R is thought to play a role in the regulation of the energy balance in fish through the modulation of feeding behavior^15^,^16^,^17^. MC5Rs are found mainly in exocrine tissues; we do not further address this receptor in this study.

Alpha-MSH is (at least in the few species studied so far) also involved in the stress response of fish^1^. In gilthead sea bream (*Sparus aurata*) and in rainbow trout (*Oncorhynchus mykiss*) air exposure, a severe stress for fish, induces an increased α-MSH levels^18^,^19^. Moreover, other studies on salmonids have shown that ACTH and α-MSH cells are differentially activated during stress: HPI axis activation by handling and confinement led to elevated plasma concentrations of ACTH only, but when these stressors were combined with a thermal shock α-MSH was also increased^20^. Similarly, the latter is supported by studies on tilapia (*Oreochromis mossambicus*): prolonged netting stress had no effect on ACTH concentrations, but plasma cortisol levels did increase suggesting activity of another corticotrope; when netting was combined with confinement both cortisol and ACTH increased^21^, and this suggests that the confinement was a second acute stressor activating the ACTH pathway. More studies have shown that MSH is a corticotrope in Mozabique tilapia^22^, in barfin flounder^23^ and in rainbow trout^24^, but not in carp (*Cyprinus carpio*)^10^. Taken together, these results do suggest a functional role for α-MSH during stress, but whether α-MSH is a corticotrope remains unclear. The search for MCRs other than the MC2R in interrenal tissue is indicated.

In teleosts, cortisol acts as glucocorticoid and mineralocorticoid (fish lack aldosterone synthase) and is the most commonly used hormonal indicator of stress^1^,^25^. The hormone binding domain in fish glucocorticoid and mineralocorticoid receptors is very similar and binds cortisol, whereas the DNA binding domains define the eventual functionality of the receptors.

Recently, the cortisol response and its molecular regulation during early ontogeny have been studied in European sea bass^26^,^27^. So far to the best of our knowledge no data in fish physiology exists about the ontogenetic pattern of α-MSH levels, the effects of a stressor on its levels during early ontogeny or the molecular mechanisms involved. To this end, we examined α-MSH temporal patterns and the expression profiles of *pomc, mc1r, mc2r* and *mc4r* genes in European sea bass during defined stages of early development; also we analyzed the response to an acute stressor prior to and after earlier exposure to stress at the end of the larval period.

## Results

### Temporal patterns of α-MSH content and gene expression at early ontogeny

**Figure 1:**
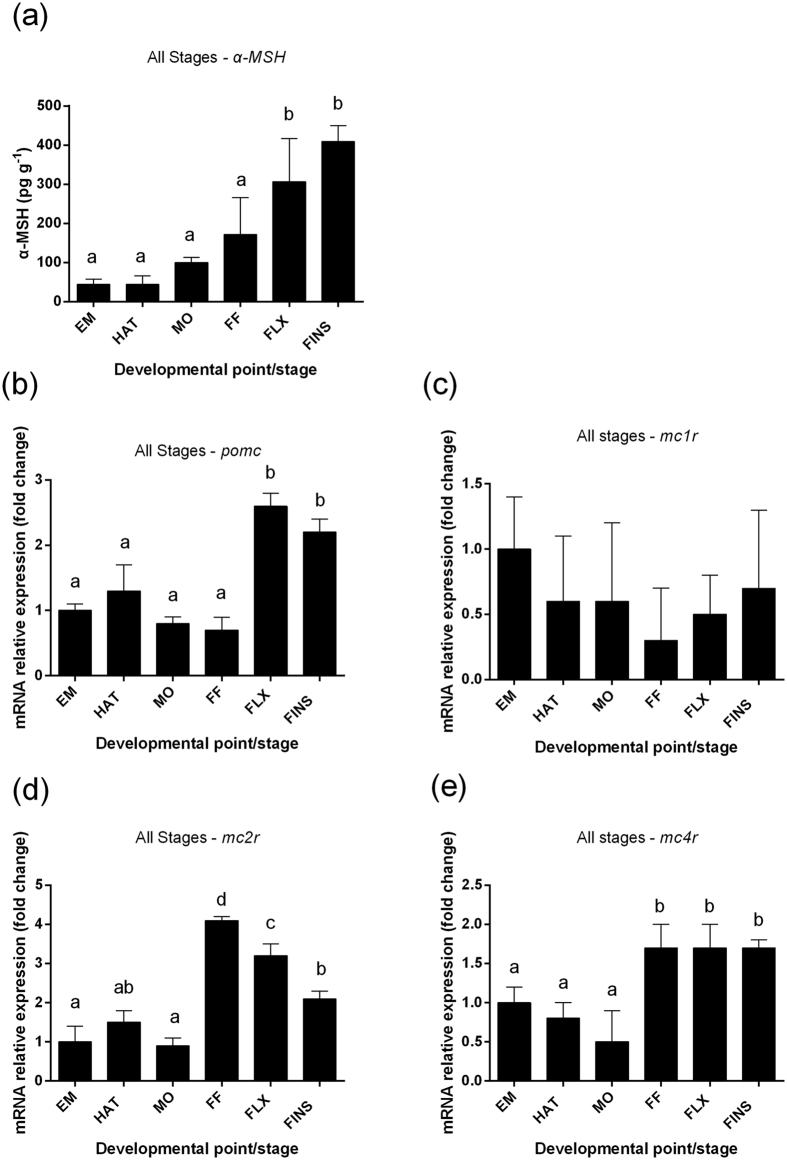
Temporal patterns of α-MSH content and gene expression at early ontogeny of European sea bass. Changes in resting whole body (**a**) α-MSH and mRNA transcript levels of (**b**) *pomc*, (**c**) *mc1r,*(**d**) *mc2r* and (**e**) *mc4r* at the different developmental points/stages (embryos-EM, hatch-HAT, mouth opening-MO, first feeding-FF, flexion-FLX, formation of all fins-FINS). Means with different letters differ significantly from one another (*P* < 0.05).

European sea bass embryos had low basal α-MSH content (44.5 ± 13.5 pg g^−1^) that subsequently increased at first feeding (172 ± 93.5 pg g^−1^), but these differences were not statistically significant. A significant increase was observed at flexion (307 ± 101 pg g^−1^) and remained at statistically significant high values onwards to the formation of all fins (408.8 ± 41.1 pg g^−1^), [Fig. 1a; (F_5,12_ = 17.22; *P* < 0.001)]. All genes of interest are expressed in all developmental stages examined (Fig. 1). Transcripts of *pomc* (Fig. 1b) showed a decrease in mRNA abundance from embryos till first feeding and a statistically significant 2,6-fold increase at the stage of flexion and remained high during ‘all fins’ (F_5,30_ = 41.13; *P* < 0.001). Expression levels of *mc1r* (Fig. 1c) showed no statistically significant differences between the developmental stages examined. Expression of *mc2r* (Fig. 1d) showed low levels from embryos till mouth opening and at the stage of first feeding there was a 4,1-fold upregulation that gradually dropped at flexion and ‘all fins’ (F_5,30_ = 47.88; *P* < 0.001). Expression levels of *mc4r* (Fig. 1e) remained low from the embryo stage until mouth opening; a statistically significant 1,8-fold increase was seen at the stage of first feeding and it remained at the same levels till the formation of all the fins (F_5,30_ = 18.28; *P* < 0.001).

### α-MSH localization

**Figure 2:**
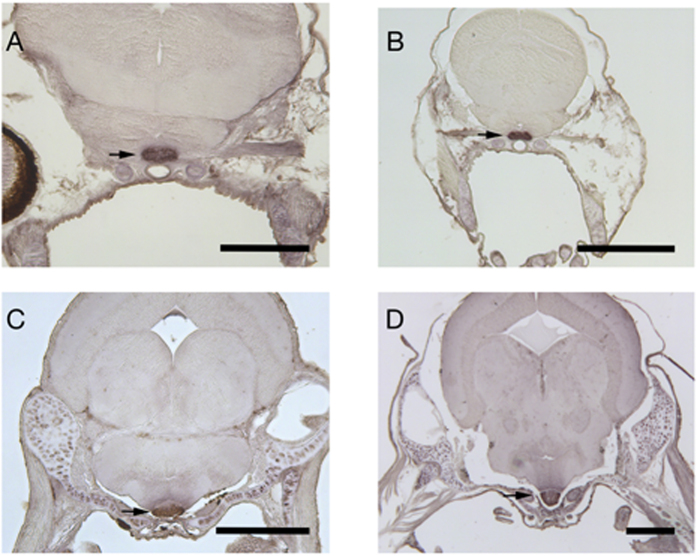
α-MSH radioimmunoassay. Representative images of transverse sections of (**A**) mouth open, (**B**) first feeding, (**C**) flexion and (**D**) all fins stages. Arrows indicate α-MSH positive cells in the *pars intermedia*. The scale bar represents 100 μm (**A**) or 200 μm (**B–D**).

Alpha-MSH positive cells in the pituitary *pars intermedia* (indicated by arrows in Fig. 2) were observed in all stages examined (mouth open, first feeding, flexion and all fins: Fig. 2A–D respectively). Omission of the primary antibody resulted in loss of all staining, which illustrates that our results show specific α-MSH staining. Besides α-MSH positive cells in the pituitary gland, a low intensity (background) staining was observed in chondrocytes. No other positive α-MSH cells were observed (data not shown).

### α-MSH and cortisol concentrations and mRNA expression levels of pomc, mc1r, mc2r and mc4r following the acute stress application

**Figure 3:**
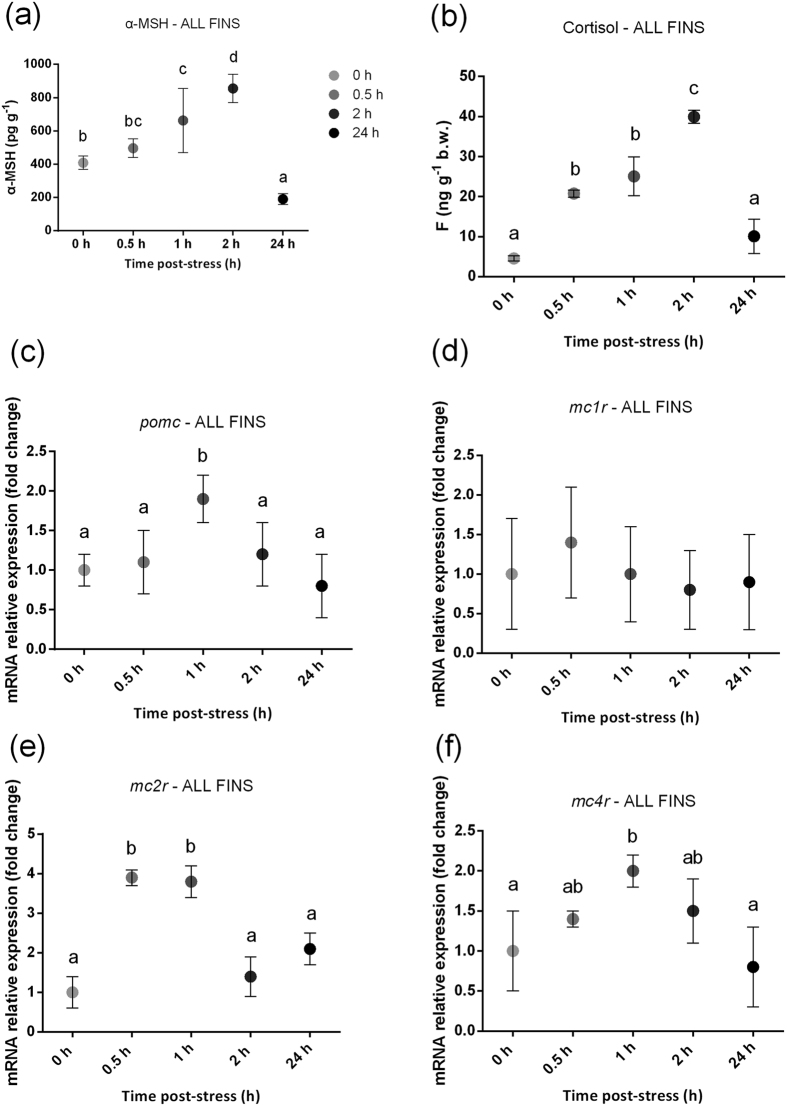
The response to stress at the stage of the formation of all the fins in *Dicentrarchus labrax*. European sea bass larvae were exposed to acute stressors and the whole body α-MSH content (**a**), whole body cortisol content (**b**) and differences in the expression levels of genes (**c**: *pomc*; **d**: *mc1r*; **e**: *mc2r*; **f**: *mc4r*) was analyzed prior to (0 h) and after (0.5 h, 1 h, 2 h and 24 h) the application of the stressor. Means with different letters differ significantly from one another (*P* < 0.05).

Figures 3 shows the α-MSH and cortisol response and the expression profile of the *pomc, mc1r, mc2r* and *mc4r* genes prior to (0 h) and after (0.5 h, 1 h, 2 h and 24 h) the application of a stressor at the stage of the formation of all fins. A statistically significant effect of the stressor on α-MSH concentrations was observed (Fig. 3a) with a pattern characterized by a gradual increase to a maximum at 2 h (855.7 ± 84.7 pg g^−1^) followed by a minimum (190.6 ± 31.9 pg g^−1^) at 24 h post stress (F_4,10_ = 19.07; *P* < 0.001). Whole-body cortisol content (Fig. 3b) was 4.58 ± 0.7 ng g^−1^ at 0 h, to increase to 20.75 ± 0.9 ng g^−1^ at 0.5 h, reach a maximum at 2 h (39.93 ± 1.6 ng g^−1^) after stress, and then returned to basal values at 24 h (10.11 ± 4.3 ng g^−1^) (F_4,10_ = 62.14; *P* < 0.001).

Transcripts levels of *pomc* (Fig. 3c) showed a statistically significant 1.9-fold upregulation at 1 h post stress compared to controls (F_4,25_ = 6.67; *P* < 0.05) that dropped to resting values at 24 h post stress. The acute stress application had no effect on *mc1r* (Fig. 3d). The pattern observed for *mc2r* (Fig. 3e) expression after the acute stress application consisted of low basal values at 0 h that increased at 0.5 h and at 1 h post stress (3.8-fold up-regulation) which returned to basal values at 2 h and 24 h post stress (F_4,25_ = 12.55; *P* < 0.001). This expression pattern of *mc2r* after the acute stress application parallels with the changes observed in whole body cortisol concentrations post stress. Transcript levels of *mc4r* (Fig. 3f) were affected by the applied stressors, showing a 2-fold up-regulation at 1 h post stress, to gradually reach basal values at 24 h post stress (F_4, 25_ = 6.78; *P* < 0.05).

## Discussion

During ontogenesis of sea bass, temporal changes in whole-body α-MSH levels showed a gradual increase from low levels during the first stages to maximum values at the stages of flexion and development of all fins. Our immunohistochemistry showed that no significant α-MSH positive cells were observed outside of the *pars intermedia*. There are very few data available on α-MSH during early ontogeny apart from a study carried out during the early developmental stages in scyliorhinid dogfish (*Scyliorhinus torazame*) that showed a gradual increase of the α-MSH-producing cells in the adenohypophysis^28^. The observed increase in whole-body α-MSH concentrations at the advanced stages of early development reported here may reflect the involvement of α-MSH in the formation of melanophores and the coloring of the body^29^,^30^,^31^,^32^, which takes place in the period around the formation of the fins. Expression of *pomc* increases at the stage of flexion and its peak is in line with the first statistically significant elevation of α-MSH levels. Abundance of *mc2r* remains at low levels until the stage of first feeding where it reaches a maximum and then decreases gradually at the later stages of development. Previous studies of our group have shown that sea bass larvae begin to synthesize cortisol around the stage of first feeding^26^,^27^, which coincides with the expression profile observed for *mc2r*. Similar results have been obtained in zebrafish, where the expression of *mc2r* is upregulated immediately before the rise in whole-body larvae cortisol concentrations^33^. Expression of *mc1r* was not altered depending on the developmental stage, whereas expression levels of *mc4r* was low in the embryo stage until mouth opening showing an increase at the stage of first feeding and remained at similar levels thereafter till the stage of the full formation of all fins. The acute stress challenge at the stage of the formation of all fins involved an elevation of α-MSH and cortisol levels with a peak at 2 h after application of the stressor. These results are supported by studies in adult gilthead sea bream (*Sparus aurata*) and rainbow trout (*Oncorhynchus mykiss*), studies that showed that the application of severe acute stress leads to an increase of α-MSH levels^18^,^19^. This pattern of the α-MSH response to stress is rather similar to the pattern observed post-stress for whole body cortisol concentrations at the same developmental stage, where low basal values increase at 0.5 h and 1 h to reach a maximum at 2 h post-stress (Fig. 3a).

α-MSH is a POMC-derived peptide, so to reveal the molecular mechanisms related to the onset of the α-MSH stress response, qPCR experiments were carried out to analyze transcript levels of *pomc.* We would emphasize that we do not – as of yet – know how the measured transcripts are reflected in peptide levels of ACTH and α-MSH. Expression levels of *pomc* after the acute stress application appear to be altered at the stage of the formation of all fins where the pattern of *pomc* stress response is characterized by maximum values at 1 h post stress that gradually drop to resting levels at 24 h (Fig. 3c) and this could concern both the ACTH and the MSH signal. However, the mRNA expression of *pomc* is upregulated along with α-MSH levels, indicating a strong relation between *pomc* mRNA expression and α-MSH production. These results are in accordance with the results obtained from a study in adult channel catfish (*Ictalurus punctatus*), where an up-regulation of *pomc* mRNA was observed in response to low-water stress, showing peak values at 1 h post stress which at 3 h declined to the level of the control group^34^.

The melanocortins exert their physiological role by binding to melanocortin receptors (MC1R-MC5R). MC2R plays a critical role in the HPI axis^35^ and is specifically activated by ACTH, while the other MCRs can be activated by the MSHs as well as ACTH^7^. The acute stress application at the stage of the formation of all fins resulted in altered transcript levels of *mc2r* which showed an up-regulation after application of the stressor, with peak values at 0.5 h and 1 h post stress to return to basal levels at 2 h and 24 h post stress (Fig. 3e) showing a similar pattern with the observed cortisol pattern obtained under the same conditions (Fig. 3b). The observed up-regulation of *mc2r* after stress at the stages of flexion and development of all fins is in accordance with the data obtained in a study conducted in rainbow trout where application of an acute stressor led to increased levels of *mc2r* transcripts^12^. Up-regulation of *mc2r* after acute stress is further supported by a study by Tokarz and colleagues in zebrafish^36^, where the expression level of *mc2r* increased significantly until about 30 min after the stressor and subsequently decreased to the mRNA levels of unstressed fish. Both *mc1r* and *mc4r* are activated by α-MSH and are involved in the control of the pigmentation and the modulation of food intake, respectively^15^,^16^,^17^. Expression of *mc1r* in this study was not altered following application of the stressor (Fig. 3d), whereas *mc4r* expression appeared to be affected by stress at the stage of the full formation of all the fins where transcript levels peaked at 1 h post stress and fell back to resting levels at 24 h post stress (Fig. 3f). It thus seems likely that MSH also through MC4R may play a role in the responses to stress.

In summary, we characterized for the first time in a Mediterranean marine teleost, the European sea bass, the temporal pattern of whole body α-MSH and the expression profile of *pomc, mc1r, mc2r* and *mc4r* genes during early ontogeny. Additionally, sea bass larvae at the stage of the full formation of all fins were exposed to acute stressors and the temporal patterns of whole body α-MSH and cortisol and the expression profiles of *pomc, mc1r, mc2r* and *mc4r* genes prior to and 0.5 h, 1 h, 2 h, and 24 h after application of the stressor, were determined. Overall, these data, combined with data on the cortisol response during early ontogeny^26^,^27^ give us for the first time a more thorough view on the two mechanisms involved in the stress response in sea bass with similar patterns observed for α-MSH and cortisol. Alpha-MSH is a truly pleiotropic hormone, with, among others, effects on skin coloration, feed intake and metabolism. To what extent α-MSH contributes to each of these processes separately in early development remains to be determined, but our results indicate an involvement of α-MSH in the stress response, a response requiring adjustment of energy flow and distribution. Whether MSH acts on the interrenal and/or on brain centers controlling feed intake remains to be determined.

## Materials and Methods

### Ethics statement

The laboratories of the Hellenic Centre for Marine Research are certified and obtained the codes for breeding animals for scientific purposes (EL-91-BIO-04). All procedures involving the handling and treatment of fish used during this study were approved by the HCMR Institutional Animal care and use committee following the Three Rs (3Rs, Replacement, Reduction, Refinement) guiding principles for ethical use of animals in testing, in accordance to Greek (PD 56/2013) and EU (Directive 63/2010) legislation on the care and use of experimental animals.

### Animals and husbandry conditions

Batches of fertilized European sea bass eggs were obtained from a private fish farm (DIAS S.A.) and transferred to the installations of the Institute of Aquaculture, Hellenic Center for Marine Research (Heraklion, Crete). Larval rearing was performed applying the pseudogreen-water technique^37^, in 500 L cylindro-conical tanks, with an initial density of 100 eggs L^−1^ in which both hatching and rearing took place. A biological filter was coupled to the tanks which were initially filled with filtered seawater from a deep well. Water, during embryogenesis, egg hatching, and at the autotrophic larval stage, was re-circulated from the bottom of the tank through the biological filter at a rate of 10% of the tank volume per h and was progressively increased to 70% of the tank volume per h until the end of the trial. Aeration (compressed air) was provided by means of a wooden diffuser located in the tank center at a rate of 150–200 ml min^−1^. Larvae were held during the whole experimental period under a mean (±SD) water temperature of 18 (±1.6) °C, dissolved oxygen levels of 7.2 ± 0.8 mg l^−1^, salinity of 36‰ and pH of 7.9–8.2. During hatching and until mouth opening, tanks were kept in complete darkness; a 12D:12 L photoperiod regime (lights on at 08:00 h) was applied during the rest of the experiment. Following mouth opening and eye development, sea bass larvae were exposed to low light intensity conditions (5–10 lux) in the absence of food for a period of 2 to 4 days to ensure normal swim bladder inflation, while the water surface was also kept free from any (food derived) oily film by the use of an air-blower skimmer. Food was delivered only when inflated swim bladder was observed in more than 80% of the population. Exogenous feeding was based on rotifers (*Brachionus* sp.) at 5 individuals ml^−1^ enriched with proteins and PUFA (INVE Aquaculture S.A., Belgium) until 10 days post hatching (dph); phytoplankton (*Chlorella* sp.) was supplied until 10 dph. Enriched *Artemia* nauplii (Instar ΙΙ, EG, Artemia Systems S.A., Belgium) were administered from 10 dph until 50 dph at 0.5 to 1.0 individual ml^−1^. From 30 dph, larvae were offered dry feed (PROTON 2–3, INVE Aquaculture S.A., Belgium) through the use of automated feeders. The trial lasted until individuals completed the formation of their fins at 45 days post hatch (dph).

### Experimental design

Samples were collected at six different points/stages during early life development (embryos, hatching, mouth opening, first feeding, flexion and formation of all fins). Additionally, at the stage of the formation of all fins samples were taken prior to and after the application of an acute stressor: the larvae were exposed to high aeration (1,000–1,500 ml min^−1^ vs. 150–200 ml min^−1^) for 90 sec, chased with a net for 20 sec, confined (collection in beakers), and air exposed for 5 sec. Samples for molecular analysis (embryos, hatched eggs and larvae samples: *n* = 6 pools of *ca.* 30 mg), cortisol (*n* = 3 pools of *ca.* 250 mg) and α-MSH (*n* = 3 pools of *ca.* 250 mg) were collected with a net, flash frozen in liquid N_2_ and stored at −80 °C until further analyses. At the stage of the formation of all fins additional samples were also collected at 0.5 h, 1 h, 2 h and 24 h post-stress.

### α-MSH radioimmunoassay

Samples were homogenized in 0.01 M HCl (1:1 (v/w) HCl/body weight). Whole-body α-MSH concentrations were then measured with a radioimmunoassay; α-MSH was labeled with ^125^I using the iodogen method^38^. Labeled α-MSH was purified by solid phase extraction (C8 Bakerbond column, J.T. Baker, Center Valley, PA, USA). The antiserum shows 100% cross reactivity with des-, mono- and di-acetyl- α-MSH^39^, and was used in a final concentration of 1:22,500. The second antibody to precipitate immune-complexes was a sheep-anti-rabbit anti-body (Fitzgerald, Acton, MA, USA) and was used at a final dilution of 1:15. Radioimmunoassay analyses using recombinant ACTH peptides demonstrated no binding of the antibody at all (0% cross-reactivity). Mammalian as well as fish ACTH peptides were used, which all share a sequence similarity between 80–95% with sea bass ACTH. Moreover, alpha-MSH is acetylated N-terminally, and the antibody shows 100% cross-reactivity between des-, mono- and di-acetyl alpha-MSH, indicating that the epitope of the antibody lies in the C-terminal region of the protein. This region is not available for antibody binding in ACTH and not present in CLIP, nor any other POMC-derived peptides. Therefore, we are confident that the antibody used in the present study is highly specific for alpha-MSH.

### α-MSH immunohistochemistry

Samples of mouth opening, first feeding, flexion and all fins were fixed in Bouin’s fixative for 6 hours and wash thoroughly in 70% ethanol afterwards. For sectioning, samples were dehydrated trough gradually increasing ethanol series and embedded in paraffin. Sections (7 μm) were cut, mounted on glass slides coated with poly-l-lysine and dried overnight at 37 °C. Next day the sections were deparaffinized in xylene, rehydrated through gradually decreasing ethanol series and incubated with 1% H_2_O_2_ to block endogenous peroxidase activity. Non-specific antigenic sites were blocked with 2% Normal Donkey Serum (NDS, Jackson Immuno Reseach, West Grove, PA) followed by overnight incubation with 1:1000 α-MSH antibody^39^. After 1 h incubation with 1:200 biotinylated Donkey Anti Rabbit IgG (Jackson Immuno Reseach, West Grove, PA) secondary antibody, 1:200 Avidin-Biotin-HRP Complex (ABC, Vector Laboratories, Burlingame, CA) was added and incubated for 1 h. Staining was performed using 0.025% 3,3′diaminobenzidine (DAB) and 0.005% H_2_O_2._

### Cortisol determination

Whole body cortisol extraction was performed according to Pavlidis *et al*.^26^. Briefly, body samples were partially thawed on ice and homogenized in 5 × (w/v), ice-cold phosphate-buffered saline (pH 7.4). Cortisol was extracted from 2 × 250 μL of homogenate with 3 mL of diethyl ether. The extract was allowed to freeze by placing tubes in a deep freezer (−80 °C), the diethyl ether layer (above the frozen water layer) was transferred into a new tube and evaporated by placement of tubes in a 45 °C water bath for 1 h and in room temperature for an additional 3 h. Samples were then reconstituted in 250 μL of immunoassay buffer and cortisol was quantified by the use of a commercial enzyme immunoassay kit (Cayman Chemical, MI, USA). All samples were tested in duplicate.

### RNA purification and cDNA synthesis

Samples of embryos, pre-larvae and larvae were let to thaw on ice, disrupted and homogenized using the TissueRuptor (Qiagen, Hilden, Germany) for 20 s in 600 μl RLT plus buffer (RNeasy Plus Mini Kit Qiagen, Valencia, USA). Total RNA was isolated with the RNeasy Plus Mini Kit (Qiagen, Valencia, USA). RNA yield and purity was determined by measuring the absorbance at 260 and 280 nm using a Nanodrop^®^ ND-1000 UV–Vis spectrophotometer (Peqlab, Erlangen, Germany), and its integrity was tested by electrophoresis in 1% agarose gels. Reverse transcription (RT) was carried out using QuantiTect Reverse transcription kit (Qiagen, Valencia, USA) using 1 μg of total RNA, according to the manufacturer’s instructions.

### Primer design for mc1r, mc2r, mc4r and pomc genes

Primers for *mc4r* were as described by Sanchez *et al*.^40^, whereas the reference genes *eukaryotic elongation factor 1* (*elf1a*) and *ribosomal 18S RNA* (*18S*) were used as in previous work^41^. Primer design for *melanocortin 1 receptor* (*mc1r*), *melanocortin 2 receptor* (*mc2r*) and *pro-opiomelanocortin* (*pomc*) was based on the available sequences with accession numbers FN377856.1^42^, FR870225.1^14^, and AY691808.1^4^. The forward primer for *mc1r* has the sequence 5′ CTCCACCTCATCCTCATC 3′ while the reverse 5′ GAAGCACCAAGAACACAG 3′. In the case of *mc2r* the forward primer has the sequence 5′ CATCTACGCCTTCCGCATTG 3′ and the reverse 5′ ATGAGCACCGCCTCCATT 3′. The forward primer for *pomc* has the sequence 5′ CCGGTCAAAGTCTTCACCTC 3′ while the reverse 5′ ACCTCCTGTGCCTTCTCCTC 3′. The products of each primer pair were further checked with sequencing to confirm amplification of the desired genes.

### Real-time quantitative PCR (qPCR)

Relative expression of *mc1r, mc2r, mc4r* and *pomc* was determined with quantitative polymerase chain reaction (qPCR) assays using the *KAPA SYBR*^®^ *FAST* qPCR Kit (Kapa Biosystems), according to the manufacturer’s instructions. The resulting fluorescence was detected with CFX Connect Thermal Cycler (Bio-Rad) under the following cycling parameters: 95 °C for 3 min, 94 °C for 15 sec, 60 °C for 30 sec (for *mc2r* and *mc4r*)/55 °C for 30 sec (for *pomc* and *mc1r*), 72 °C for 20 sec, 40 cycles. Levels of *mc1r, mc2r, mc4r* and *pomc* mRNA were normalized using reference genes *18S* and *elf1a*. A standard curve was constructed for each gene, using 4 serial dilutions (1:5) of a pool of all cDNA samples by graphing the negative log of the dilution factor against the relative cycle threshold value. To be considered suitable for analysis, each primer pair was required to have a linear standard curve with an r^2^ value above 0.98 and primer amplification efficiency between 90% and 100%. We performed geNORM analysis^43^ to validate the reference genes that served as internal control, by defining a gene-stability measure *M* which corresponds to the standard deviation of the logarithmically transformed expression values of the compared genes. This stability index together with its decreasing value gives rise to the rank and stability of each gene. In our study *18S* and *elf1a* genes proved have the most stable expression (target stability value *M* < 0.5) and the mean of their normalization factors was used for each sample.

### Statistical analysis

All statistical analyses were performed with SigmaPlot 11.0 (Jandel Scientific). Data are presented as means ± standard deviation (SD). Statistical comparisons of α-MSH concentration and gene expression of unstressed specimens (0 h) between the different developmental points/stages and statistical comparisons of temporal patterns of α-MSH and cortisol concentrations and gene expression between the different time points following exposure to a stressor at the stage of the formation of all fins were made using one-way ANOVA. Holm-Sidak’s honestly significant difference test for multiple comparisons was used to determine significant differences among groups. The significance level was set at *P* < 0.05.

## Additional Information

**How to cite this article:** Tsalafouta, A. *et al*. a-MSH and melanocortin receptors at early ontogeny in European sea bass (*Dicentrarchus labrax,* L.). *Sci. Rep.* **7**, 46075; doi: 10.1038/srep46075 (2017).

**Publisher's note:** Springer Nature remains neutral with regard to jurisdictional claims in published maps and institutional affiliations.

## References

[b1] Wendelaar Bonga S. E. The stress response in fish. Physiol Rev 77, 591–625 (1997). 9234959 10.1152/physrev.1997.77.3.591

[b2] Slominski A., Wortsman J., Luger T., Paus R. & Solomon S. Corticotropin releasing hormone and proopiomelanocortin involvement in the cutaneous response to stress. Physiol. Rev. 80, 979–1020 (2000). 10893429 10.1152/physrev.2000.80.3.979

[b3] Smith A. I. & Funder J. W. Proopiomelanocortin processing in the pituitary, central nervous system, and peripheral tissues. Endocr. Rev. 9, 159–179 (1988). 3286233 10.1210/edrv-9-1-159

[b4] Varsamos S., Wendelaar Bonga S. E., Flik G., Queré R. & Commes T. Cloning of a proopiomelanocortin cDNA from the pituitary gland of the sea bass (*Dicentrarchus labrax*) and assessment of mRNA expression in different tissues by means of real-time PCR. J. Endcrinol. 176, 405–414 (2003). 10.1677/joe.0.1760405 12630925

[b5] Metz J. R., Peters J. J. M. & Flik G. Molecular biology and physiology of the melanocortin system in fish: a review. Gen. Comp. Endocrinol. 148, 150–162 (2006). 16620815 10.1016/j.ygcen.2006.03.001

[b6] Cerdá-Reverter J. M. . Fish Melanocortin System. Eur. J. Pharmacol. 660, 53–60 (2011). 21208603 10.1016/j.ejphar.2010.10.108

[b7] Schiöth H. B. . Evolutionary conservation of the structural, pharmacological and genomic characteristics of the melanocortin receptors subtypes. Peptides 26, 1886–1900 (2005). 15985310 10.1016/j.peptides.2004.11.034

[b8] Barton B. A. Stress in fishes: a diversity of responses with particular reference to changes in circulating corticosteroids. Integr. Comp. Biol. 42, 517–525 (2002). 21708747 10.1093/icb/42.3.517

[b9] Klovins J. . The melanocortin system in Fugu: determination of POMC/AGRP/MCR gene repertoire and synteny, as well as pharmacology and anatomical distribution of the MCRs. Mol. Biol. Evol. 21, 563–579 (2004). 14694081 10.1093/molbev/msh050

[b10] Metz J. R., Geven E. J., van den Burg E. H. & Flik G. ACTH, alpha- MSH, and control of cortisol release: cloning, sequencing, and functional expression of the melanocortin-2 and melanocortin-5 receptor in *Cyprinus carpio*. Am. J. Physiol. 289, 814–826 (2005). 10.1152/ajpregu.00826.2004 15890786

[b11] Agulleiro M. J. . Role of accessory proteins in the function of zebrafish melanocortin receptor type 2. Mol. Cell. Endocrinol. 320, 145–152 (2010). 20138960 10.1016/j.mce.2010.01.032

[b12] Aluru N. & Vijayan M. M. Molecular characterization, tissue-specific expression, and regulation of melanocortin 2 receptor in rainbow trout. Endocrinology 149, 4577–4588 (2008). 18535097 10.1210/en.2008-0435 PMC2553378

[b13] Liang L., Schmid K., Sandhu N., Angleso J. K., Vijayan M. M. & Dores R. M. Structure/function studies on the activation of the rainbow trout melanocortin-2 receptor. Gen. Comp. Endocrinol. 210, 145–151 (2015). 24709361 10.1016/j.ygcen.2014.03.032

[b14] Agulleiro M. J. . Molecular characterization and functional regulation of Melanocortin 2 Receptor (MC2R) in the sea bass. A putative role in the adaptation to stress. PLoS ONE 8(5), e65450 (2013). 23724142 10.1371/journal.pone.0065450 PMC3664627

[b15] Cerdá-Reverter J. M., Ringholm A., Schiöth H. B. & Peter R. E. Molecular cloning, pharmacological characterization, and brain mapping of the melanocortin 4 receptor in the goldfish: involvement in the control of food intake. Endocrinology 144, 2336–2349 (2003a). 12746294 10.1210/en.2002-0213

[b16] Cerdá-Reverter J. M., Schiöth H. B. & Peter R. E. The central melanocortin system regulates food intake in goldfish. Regul. Pept. 115, 101–113 (2003b). 12972325 10.1016/s0167-0115(03)00144-7

[b17] Song Y. & Cone R. D. Creation of a genetic model of obesity in a teleost. Faseb. J. 21, 2042–2049 (2007). 17341684 10.1096/fj.06-7503com

[b18] Arends R. J., Mancera J. M., Muñoz J. L., Wendelaar Bonga S. E. & Flik G. The stress response of the gilthead sea bream (*Sparus aurata* L.) to air exposure and confinement. J. Endocrinol. 163, 149–157 (1999). 10495417 10.1677/joe.0.1630149

[b19] Sumpter J. P., Dye H. M. & Benfey T. J. The effects of stress on plasma ACTH, a-MSH and cortisol levels in salmonid fishes. Gen. Comp. Endocrinol. 62, 377–385 (1986). 3021561 10.1016/0016-6480(86)90047-x

[b20] Sumpter J. P., Pickering A. D. & Pottinger T. G. Stress-induced elevation of plasma alpha-MSH and endorphin in brown trout, *Salmo trutta L*. Gen. Comp. Endocrinol. 59, 257–265 (1985). 4040489 10.1016/0016-6480(85)90377-6

[b21] Balm P. H. M., Pepels P., Helfrich S., Hovens M. L. & Wendelaar Bonga S. E. Adrenocorticotropic hormone in relation to interrenal function during stress in tilapia (*Oreochromis mossambicus*). Gen. Comp. Endocrinol. 96, 347–360 (1994). 7883141 10.1006/gcen.1994.1190

[b22] Lamers A. E., Flik G., Atsma W. & Wendelaar Bonga S. E. A role for di-acetyl alpha-melanocyte-stimulating hormone in the control of cortisol release in the teleost *Oreochromis mossambicus*. J. Endocrinol. 135, 285–292 (1992). 1335471 10.1677/joe.0.1350285

[b23] Kobayashi Y., Chiba H., Yamanome T., Schiöth H. B. & Takahashi A. Melanocortin receptor subtypes in interrenal cells and corticotropic activity of a-melanocyte-stimulating hormones in barfin flounder, Verasper moseri. Gen. Comp. Endocrinol. 170, 558–568 (2011). 21118693 10.1016/j.ygcen.2010.11.019

[b24] Rance T. A. & Baker B. I. The *in vitro* response of the trout interrenal to various fragments of ACTH. Gen. Comp. Endocrinol. 45, 497–503 (1981). 6277731 10.1016/0016-6480(81)90054-x

[b25] Barton B. A. & Iwama G. K. Physiological changes in fish from stress in aquaculture with emphasis on the response and effects of corticosteroids. Ann. Rev. Fish. Dis. 1, 3–26 (1991).

[b26] Pavlidis M. . Onset of the primary stress in European sea bass *Dicentrarchus labrax*, as indicated by whole body cortisol in relation to glucocorticoid receptor during early development. Aquaculture 315, 125–130 (2011).

[b27] Tsalafouta A. . Ontogenesis of the HPI axis and molecular regulation of the cortisol stress response during early development in *Dicentrarchus labrax*. Sci. Rep. 4, doi: 10.1038/srep05525 5525 (2014). 24984570 PMC4078316

[b28] Chiba A. & Oka S. Ontogenetic changes of alpha-melanocyte-stimulating hormone (*α*-MSH)-like immunoreactivity in the brain and hypophysis of a scyliorhinid dogfish (*Scyliorhinus torazame*): An immunohistochemical study. Fish. Physiol. Biochem. 25, 165–170 (2002).

[b29] Eberle A. N. The Melanotropins. Karger, Basel (1988).

[b30] Fujii R. & Oshima N. Control of chromatophore movements in teleost fishes. Zool. Sci. 3, 13–47 (1986).

[b31] Fujii R. & Oshima N. Factors influencing motile activities of fish chromatophores. In: Arpigny J. L., Coyette J., Davail S., Feller G., Fonze E. (Eds) Advances in Comparative and Environmental, vol 20, Springer-Verlag, Berlin, pp. 1–54 (1994).

[b32] Lu D., Chen W. & Cone R. D. Regulation of melanogenesis by the MSH receptor. In: Nordlund J. J., Boissy R. E., Hearing V. J., King R. A., Ortonne J. P. (Eds) Pigmentary System. Physiology and Pathophysiology, Oxford Univ. Press, New Year, pp. 183–197 (1998).

[b33] Alsop D. & Vijayan M. Development of the corticosteroid stress axis and receptor expression in zebrafish. Am. J. Physiol. Integr. Comp. Physiol. 294, 711–719 (2008). 10.1152/ajpregu.00671.2007 18077507

[b34] Karsi A., Waldbieser G. C., Small B. C. & Wolters W. R. Genomic structure of the proopiomelanocortin gene and expression during acute low-water stress in channel catfish. Gen. Comp. Endocrinol. 143, 104–112 (2005). 16061068 10.1016/j.ygcen.2005.03.005

[b35] Dores R. M. & Garcia Y. Views on the co-evolution of the melanocortin-2 receptor, MRAPs, and the hypothalamus/pituitary/adrenal-interrenal axis. Molecular and Cellular Endocrinology 408, 12–22 (2015). 25573240 10.1016/j.mce.2014.12.022

[b36] Tokarz J., Norton W., Moller G., Hrabe de Angelis M. & Adamski J. Zebrafish 20b-hydroxysteroid dehydrogenase type 2 is important for glucocorticoid catabolism in stress response. PLoS ONE 8(1), e54851 (2013). 23349977 10.1371/journal.pone.0054851 PMC3551853

[b37] Papandroulakis N., Divanach P., Anastasiadis P. & Kentouri M. The pseudo-green water technique for intensive rearing of sea bream (*Sparus aurata*) larvae. Aquacult. Int. 9, 205–216 (2002).

[b38] Salacinski P. R., McLean C., Sykes J. E., Clement-Jones V. V. & Lowry P. J. Iodination of proteins, glycoproteins, and peptides using a solid-phase oxidizing agent, 1,3,4,6-tetrachloro-3 alpha,6 alpha-diphenyl glycoluril (Iodogen). Anal. Biochem. 117, 136–146 (1981). 7316186 10.1016/0003-2697(81)90703-x

[b39] Van Zoest I. D., Heijmen P. S., Cruijsen P. M. & Jenks B. G. Dynamics of background adaptation in *Xenopus laevis*: role of catecholamines and melanophore-stimulating hormone. Gen. Comp. Endocrinol. 76, 19–28 (1989). 2599346 10.1016/0016-6480(89)90028-2

[b40] Sanchez E. . Phosphodiesterase inhibitor-dependent inverse agonism of agouti-related protein on melanocortin 4 receptor in sea bass (Dicentrarchus labrax). Am. J. Physiol.-Reg. I 296, 1293–1306 (2009). 10.1152/ajpregu.90948.2008 PMC2689838 19225141

[b41] Kollias S., Fernandes J. M. O., Papandroulakis N. & Pavlidis M. Development of a chronic mild stress (CMS) model in fish for the study of the neuroendocrine mechanisms of stress in European sea bass (*Dicentrarchus labrax).* 9^th^ International Congress on the Biology of Fish. Barcelona, 5–9 July, Spain (2010).

[b42] Sanchez E., Rubio V. C. & Cerda-Reverter J. M. Molecular and pharmacological characterization of the melanocortin type 1 receptor in the sea bass. Gen. Comp. Endocrinol. 165, 163–169 (2010). 19539622 10.1016/j.ygcen.2009.06.008

[b43] Vandesompele J. . Accurate normalization of real-time quantitative RT-PCR data by geometric averaging of multiple internal control genes. Genome Biol. **3**, RESEARCH0034 (2002). 10.1186/gb-2002-3-7-research0034 PMC126239 12184808

